# Memory T Cells in Latent *Mycobacterium tuberculosis* Infection Are Directed against Three Antigenic Islands and Largely Contained in a CXCR3^+^CCR6^+^ Th1 Subset

**DOI:** 10.1371/journal.ppat.1003130

**Published:** 2013-01-24

**Authors:** Cecilia S. Lindestam Arlehamn, Anna Gerasimova, Federico Mele, Ryan Henderson, Justine Swann, Jason A. Greenbaum, Yohan Kim, John Sidney, Eddie A. James, Randy Taplitz, Denise M. McKinney, William W. Kwok, Howard Grey, Federica Sallusto, Bjoern Peters, Alessandro Sette

**Affiliations:** 1 La Jolla Institute for Allergy and Immunology, La Jolla, California, United States of America; 2 Institute for Research in Biomedicine, Bellinzona, Switzerland; 3 Benaroya Research Institute, Seattle, Washington, United States of America; 4 Antiviral Research Centre, University of California, San Diego, San Diego, California, United States of America; University of Medicine and Dentistry of New Jersey, United States of America

## Abstract

An understanding of the immunological footprint of *Mycobacterium tuberculosis* (MTB) CD4 T cell recognition is still incomplete. Here we report that human Th1 cells specific for MTB are largely contained in a CXCR3^+^CCR6^+^ memory subset and highly focused on three broadly immunodominant antigenic islands, all related to bacterial secretion systems. Our results refute the notion that secreted antigens act as a decoy, since both secreted proteins and proteins comprising the secretion system itself are targeted by a fully functional T cell response. In addition, several novel T cell antigens were identified which can be of potential diagnostic use, or as vaccine antigens. These results underline the power of a truly unbiased, genome-wide, analysis of CD4 MTB recognition based on the combined use of epitope predictions, high throughput ELISPOT, and T cell libraries using PBMCs from individuals latently infected with MTB.

## Author Summary

*Mycobacterium tuberculosis* is one of the most life-threatening pathogens of all time, having infected one-third of the present human population. There is an urgent need for both novel vaccines and diagnostic strategies. Here, we were able to identify the targets most dominantly recognized by latently infected individual that successfully contain infection. These targets are contained in three broadly genomic antigenic islands, all related to bacterial secretion systems and composed by several distinct ORFs. Thus, our results suggest that vaccination with one or few defined antigens will fail to replicate the response associated with natural immunity. Our analysis also pinpoints that the Th1 cells dominating the response are associated with novel and well-defined phenotypic markers, suggesting that the response is molded by unique MTB associated factors. This study demonstrates further that the approach combining peptide binding predictions with modern high throughput techniques is generally applicable to the study of immunity to other complex pathogens. Together, our data provide a new angle in the worldwide fight against *M. tuberculosis* and could be used for diagnostic or vaccine developments.

## Introduction

Tuberculosis is one of the major causes of death from infectious disease. Current diagnostics do not distinguish active and latent infection, and the only available vaccine has limited efficacy. Hence, there is an urgent need for both novel vaccines and diagnostic strategies.

Human T cell responses to MTB involve CD4, CD8, CD1 and γ∂ T cells. Seminal studies showed that human memory T helper 1 (Th1) cells directed against the purified protein derivative (PPD) of MTB secreted IFN-γ [1]. IFN-γ has an essential role in the protective immunity to mycobacteria, as individuals with genetic defects in the IFN-γ receptor has an increased susceptibility to mycobacteria [2]. Th1 cells mainly express the chemokine receptors CCR5 and CXCR3 [3], while Th17 cells co-express CCR6 and CCR4 and Th22 cells co-express CCR6 and CCR10 [4], [5].

While several studies have reported the identification of MTB antigens, from abundant or easily purified proteins [6], [7], a truly genome-wide study to identify antigens is lacking. In only a few cases have techniques allowing ex vivo detection and/or characterization of MTB-specific T cells, prior to any *in vitro* expansion and manipulations, been utilized [8], [9], [10].

A key issue relating to MTB immunity is whether different classes of antigens elicit responses that have the same or diverse functional characteristics. MTB antigens described so far are predominantly secreted MTB proteins [11], Some of which are not essential for bacterial survival [12]. As a result, it was hypothesized that secreted proteins might act as decoy antigens, diverting the immune response from recognizing more relevant MTB proteins [13].

In this regard, two intriguing MTB protein categories are the PE/PPE proteins, and the Esx protein family, which have been shown to elicit B and T cell responses [14], [15]. The function(s) of PE/PPE proteins are not fully understood but data indicates that they influence antigen presentation and host cell apoptosis [15]. The PE/PPE genes encode almost 200 proteins (4% of the total open reading frames (ORFs)) [16], unique to Mycobacteria and most prevalent in pathogenic strains. While PE/PPE proteins are mainly located within the bacterial cell wall and cell surface, some are also secreted [17], [18].

PE/PPE genes are closely related to the Esx regions [19]. These regions encode Type VII secretion systems (T7SS), also known as Esx secretion systems. Five related, but functionally distinct and non cross-complementing T7SS (Esx 1–5), are present in MTB [20]. The best characterized is Esx-1, which encodes the Rv3874 (culture filtrate protein 10 kDa, CFP10) and Rv3875 (early secretory antigenic target 6 kDa, ESAT-6) antigens [20]. The genes encoding the Esx proteins, are arranged in tandem pairs (EsxA-W) at 11 genomic loci [21]. Esx secreted proteins have been detected from Esx-1, -3 and -5 indicating that these are functional secretion systems [22].

T cell epitopes have been described from all main MTB protein categories, indicating that protein function or cellular location per se does not determine which proteins can be recognized. Previous studies in complex pathogen systems demonstrated that immune responses are directed against a relatively large fraction of the genome [23], [24]. However, epitope reactivity is currently described only from about 4% of the approximately 4,000 ORFs of the MTB genome ([11] (IEDB, www.iedb.org)). Hence, we hypothesized that a genome-wide probe of the immunogenicity of MTB ORFs would reveal a large number of novel antigens. Defining the breadth of responses is key for the design of vaccination strategies that mirror natural immunity [25], evaluation of disease the performance of vaccine candidates and the development of diagnostics.

By combining HLA class II peptide binding predictions with modern high throughput techniques such as ex vivo ELISPOT analysis, HLA class II multimers, and the screening of T cell libraries [26], we were able for the first time to identify and characterize the genome-wide antigen response in latently infected individuals.

## Results

### Breadth and Dominance of a Genome-wide Library of MTB-derived Predicted HLA Class II Epitopes in LTBI Donors

Protein sequences from five complete MTB genomes (CDC1551, F11, H37Ra, H37Rv and KZN 1435) and sixteen draft assemblies from the NCBI Protein database (Table S1) were aligned. The binding capacity of all possible 15-mer peptides (n = 1,568,148) was predicted for 22 HLA DR, DP and DQ class II alleles (Figure S1 and Table S2) most commonly expressed in the general population [27], to select peptides predicted to bind multiple HLA class II alleles (promiscuous epitopes). This approach identifies the most dominant and prevalent responses, corresponding to approximately 50% of the total overall response [27].

A total of 20,610 peptides (with a range of 2 to 10 per ORF, and an average of 5), including 1,660 variants not totally conserved amongst the genomes considered in the analysis, were synthesized and arranged into 1,036 peptide pools of 20 peptides (Figure S1). The ex vivo production of IFN-γ by PBMCs from 28 LTBI donors induced by each of the 1,036 pools was measured utilizing ELISPOT. Pools recognized by ≥10% of donors were deconvoluted, and 369 individual MTB epitopes were identified (Table S3). Individual donors recognized, on average, 24 epitopes, underlining the large breadth of response to MTB.

**Figure 1:**
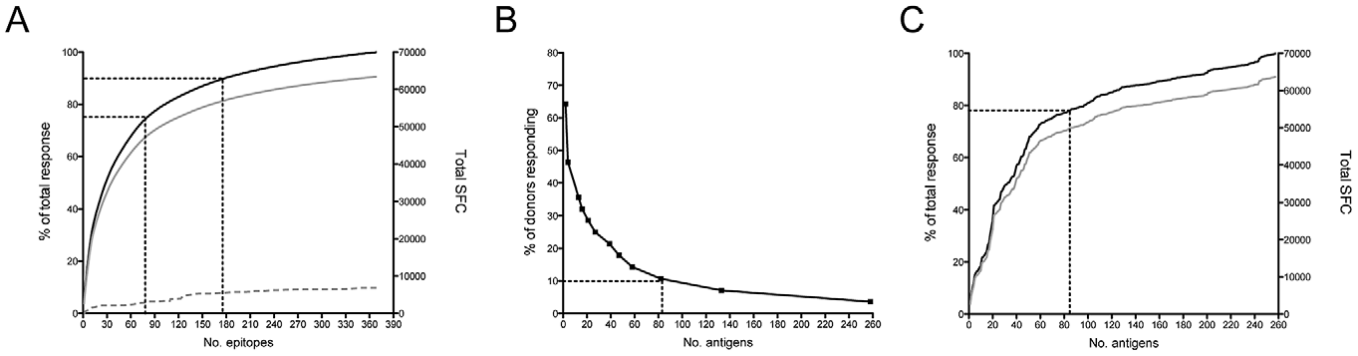
Breadth and dominance at the epitope and antigen level. (A) Epitopes ranked on the basis of magnitude of response. LTBI (black line - % of total response, grey line – total SFC) and TB uninfected (grey dashed line – total SFC) donors. Black dashed lines indicate the top 80 and 175 epitopes. (B) Antigens ranked on the basis of the response frequency for LTBI donors. Black dashed line indicates antigens recognized by >10% of LTBI donors. (C) Antigens ranked on the basis of magnitude of response and response frequency (black line - % of total response, grey line – total SFC). Black dashed line indicates the top 82 antigens.

Epitope responses were ranked on the basis of magnitude to assess their relative dominance. The top 80 epitopes accounted for 75% of the total response and the top 175 epitopes accounted for 90% of the total response (Figure 1A). Only occasional weak responses were detected in 28 TB uninfected/non-Bacille Calmette-Guérin (BCG) vaccinated control donors, thus demonstrating that these responses were LTBI-specific (Figure 1A). The epitopes were mapped to individual MTB antigens using the H37Rv as a reference genome. A total of 82 antigens were recognized by more than 10% of LTBI donors (Figure 1B). These 82 antigens accounted for approximately 80% of the total response in LTBI donors (Figure 1C). Responses to the epitopes from the most frequently recognized antigens were further characterized utilizing PBMCs depleted of either CD4 or CD8 T cells. The majority (97%) of these epitopes were recognized exclusively by CD4 T cells (Table S3), as expected because of their identification on the basis of predicted HLA class II binding capacity.

### Novel MTB Antigens and Sources of CD4 T Cell Epitopes Recognized by LTBI Donors

Comparing these 82 most prevalently recognized antigens with antigens for which similar ex vivo epitope reactivity has been described (IEDB), we found that the majority (61/82 antigens, 74%) was novel. While a given antigen might not have been analyzed in sufficient detail to lead to the description of defined epitopes, it might nevertheless have been described as a target of T cell responses. Therefore we performed a literature search for each individual antigen to further categorized them as novel, or as targets of CD4 T cells, CD8 T cells or undefined T cell type. This revealed that 41% of the antigens we identified had not previously been described as T cell targets (Figure S2A and Table 1). The responses to novel antigens, in terms of both response frequency and magnitude, are comparable to those directed against previously known T cell targets (Table S4).

**Table 1:** Summary of characteristics of novel CD4 T cell antigens recognized by more than 10% of LTBI donors, ranked according to response frequency.

Rv-number	Resp. freq.	Total SFC	Protein category	Location	T7SS
Rv3024c	32%	1630	Information pathways	Island 2	-
Rv0289	29%	2298	Cell wall and cell processes	Island 1	Esx-3
Rv0290	29%	1552	Cell wall and cell processes	Island 1	Esx-3
Rv3330	29%	1595	Cell wall and cell processes	Non-island	-
Rv1788	25%	347	PE/PPE	Non-island	Esx-5
Rv1791	25%	355	PE/PPE	Non-island	Esx-5
Rv3125c	21%	125	PE/PPE	Non-island	-
Rv0294	18%	1368	Intermediary metabolism and respiration	Island 1	-
Rv2874	18%	798	Intermediary metabolism and respiration	Non-island	-
Rv3022c	18%	109	PE/PPE	Island 2	-
Rv3135	18%	317	PE/PPE	Non-island	-
Rv3876	18%	1323	Cell wall and cell processes	Island 3	Esx-1
Rv0124	14%	177	PE/PPE	Non-island	-
Rv0291	14%	1153	Intermediary metabolism and respiration	Island 1	Esx-3
Rv0292	14%	708	Cell wall and cell processes	Island 1	Esx-3
Rv0293c	14%	1073	Conserved hypotheticals	Island 1	-
Rv0297	14%	154	PE/PPE	Non-island	-
Rv0299	14%	467	Conserved hypotheticals	Non-island	-
Rv3012c	14%	233	Information pathways	Non-island	-
Rv3025c	14%	423	Intermediary metabolism and respiration	Island 2	-
Rv0278c	11%	45	PE/PPE	Non-island	-
Rv0279c	11%	45	PE/PPE	Non-island	-
Rv0298	11%	783	Conserved hypotheticals	Non-island	-
Rv0442c	11%	232	PE/PPE	Non-island	-
Rv0690c	11%	233	Conserved hypotheticals	Non-island	-
Rv0985c	11%	70	Cell wall and cell processes	Non-island	-
Rv0987	11%	133	Cell wall and cell processes	Non-island	-
Rv1172c	11%	237	PE/PPE	Non-island	-
Rv1243c	11%	114	PE/PPE	Non-island	-
Rv1317c	11%	97	Information pathways	Non-island	-
Rv1366	11%	308	Conserved hypotheticals	Non-island	-
Rv1441c	11%	86	PE/PPE	Non-island	-
Rv2490c	11%	64	PE/PPE	Non-island	-
Rv2853	11%	85	PE/PPE	Non-island	-

Further analysis of the IEDB data revealed a limited overlap, (18%; 28/158) between antigens identified in this study and antigens known as sources of HLA class I epitopes (Figure S2B). Finally, no significant correlation was found with the antigens recognized by serological responses from the MTB proteome [28] (Figure S2C).

### HLA Class II Reactivity Is Highly Focalized on MTB Antigenic Islands

**Figure 2:**
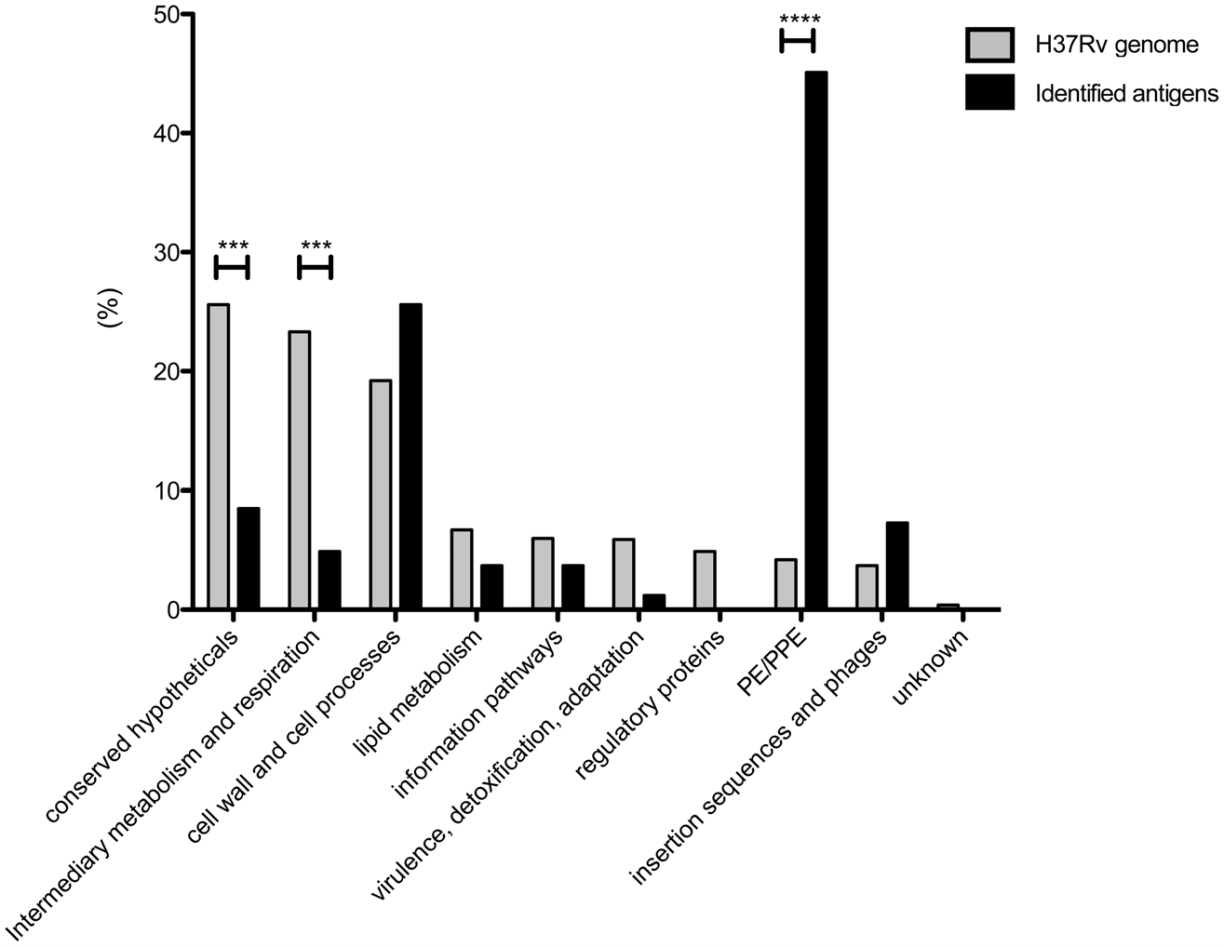
Protein categories of identified antigens. The identified antigens (black bars) were divided into protein categories (TubercuList) and compared to the MTB genome (grey bars). Chi-square test, ***, p<0.001, ****, p<0.0001.

Next, using the TubercuList database [16], we determined the protein category to which the identified antigens belong (Figure 2). As expected, the identified antigens were associated with almost every category, with the exception of regulatory proteins and proteins of unknown function. The significant overrepresentation of PE/PPE proteins was notable, as well as the underrepresentation of proteins in the conserved hypotheticals, cellular metabolism and respiration categories.

**Figure 3:**
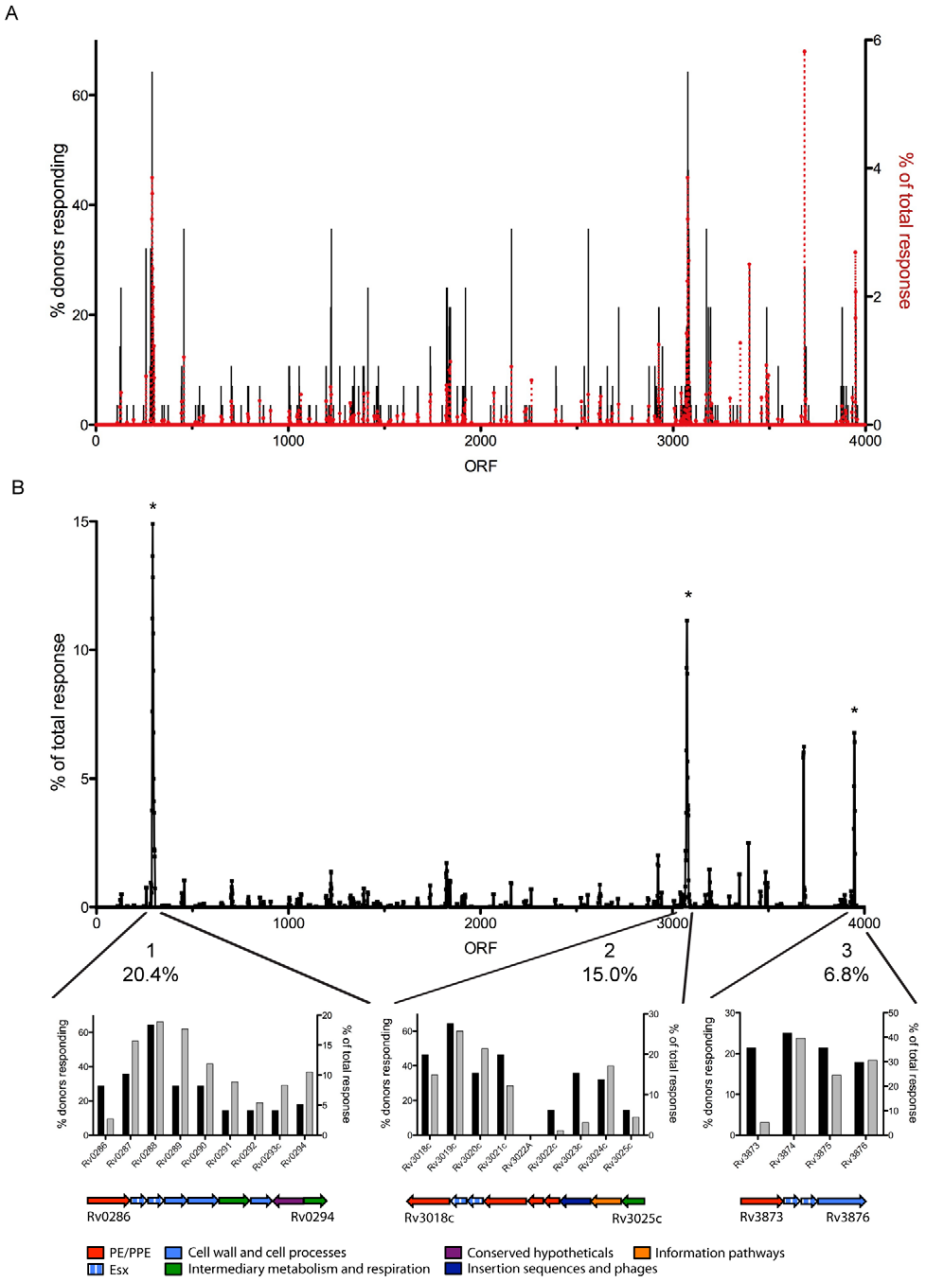
Antigens cluster in antigenic islands in the MTB genome. (A) All antigens recognized on the H37Rv genome map, % donors responding (black bars) and % of total response (red dotted line). (B) Antigenic islands identified by a 5-gene window spanning the entire MTB genome (top panel); Binomial distribution and Bonferroni correction, *, p<0.01. Proteins within each antigenic island, % donors responding (black bars) and % of total island response (grey bars) and the % of total (all antigens recognized) response per island (middle panel). Cartoons show relative length of proteins, direction of transcription and protein category of each protein. Esx proteins are part of the cell wall and cell processes category.

The localization of antigens recognized was next visualized by plotting the recognition data on a linear map of the MTB genome. Analysis of either percent of donors responding or percent of total response revealed striking clusters of reactivity within certain regions of the genome (Figure 3A). When the MTB genome was parsed into 5-gene windows, significant antigenic clusters (defined by minimum 4 proteins within the 5-gene window being recognized by 7.1% of LTBI donors) could be identified using binomial distribution probability and Bonferroni correction. Three significant antigenic islands (Figure 3B), encoding 0.55% of the total ORFs, accounted for 42% of the total response (Table 2). One of the islands (Island 3) contains the well-known Rv3875 and Rv3874 antigens, which is an Esx protein pair secreted via a T7SS. Strikingly, the other two islands also contain Esx protein pairs. Moreover, two of the antigenic islands are part of the known T7SS systems Esx-1 (Island 3) and Esx-3 (Island 1). It is noteworthy that the proteins recognized included not only the proteins believed to be secreted, but also the proteins forming the actual secretion apparatus (Island 1). Indeed, the antigens identified within these islands correspond to proteins from several different protein categories, mostly assigned to the cell wall and cellular processes and the PE/PPE category, which is not surprising since several of these proteins are part of the T7SS. Additionally, Rv3615c [29], which is functionally linked to Esx-1 [30], was also prevalently recognized. However, it stands as a single antigen and not as part of an antigenic island.

**Table 2:** Immunodominance of islands, PE/PPE, Esx and T7SS proteins.

	% donors responding	% of total response	No. proteins	% of total MTB genome	% Enrichment (% response/% genome)
**Islands total**	89	42.2	22	0.55	76.7
*Island 1*	*79*	*20.4*	*9*	*0.23*	*88.7*
*Island 2*	*86*	*15.0*	*9*	*0.23*	*65.2*
*Island 3*	*50*	*6.8*	*4*	*0.10*	*68.0*
**PE/PPE total**	71	19.2	38	0.95	20.2
*PE/PPE non-island*	*71*	*14.0*	*32*	*0.80*	*17.5*
*PE/PPE island*	*46*	*5.2*	*6*	*0.15*	*34.6*
**Esx proteins** a **total**	75	19.6	11	0.28	70.0
*Esx proteins non-island*	*11*	*1.2*	*5*	*0.13*	*9.2*
*Esx proteins island*	*75*	*18.5*	*6*	*0.15*	*123.3*
**T7SS** b **total**	79	34.7	16	0.40	86.8
*T7SS non-island*	*39*	*7.0*	*6*	*0.15*	*46.6*
*T7SS island*	*75*	*27.7*	*10*	*0.25*	*110.8*
**Other**	82	14.2	23	0.58	24.5

a ) Esx proteins include EsxA-W.

b ) T7SS includes the Esx proteins.

### Antigenic Islands Rather than PE/PPE and Esx Proteins Are the Major Determinant of Immunodominance

To dissect whether the main determinant of immunodominance was related to a given antigen being contained within an antigenic island or belonging to PE/PPE and Esx proteins families, we calculated the percentage of the total response for different groups of proteins as well as the percentage of the MTB genome associated with these protein groups (Table 2). To compare different protein groups we calculated the ratio between % of response and % genome, as a percent enrichment.

The PE/PPE proteins were responsible for 19% of the total response, and when divided into PE/PPE proteins within an island compared to non-island, the island PE/PPE were more predictive of immunogenicity than the non-island ones (Table 2). Also, in the case of Esx proteins and T7SS, proteins within the antigenic islands were more likely to be immunogenic than those outside the islands. Proteins not in the antigenic islands, and not belonging to PE/PPE and T7SS categories, were responsible for 14% of the total response (Table 2). Thus, these data show that the antigenic islands identified are highly predictive of immunogenicity, and that to be contained within the antigenic islands is the most reliable predictor of the immunodominance of PE/PPE and Esx proteins.

### Similar Multifunctionality of T Cell Responses to Different Categories of MTB Antigens

It has been proposed that some of the responses against secreted MTB proteins act as decoys [13], thereby supporting bacterial persistence. It has also been proposed that T cells differing in their degree of multifunctionality might differ in terms of protective potential, or have a role in pathology [31], [32], [33], [34]. Definition of dominant antigens allows testing the validity of these hypotheses. To address these issues we detailed responses against PE/PPE, Esx and other proteins expressed in the three major antigenic islands, or elsewhere, by a variety of approaches, including multiparameter intracellular cytokine staining (ICS) assays, tetramer staining and T cell libraries.

**Figure 4:**
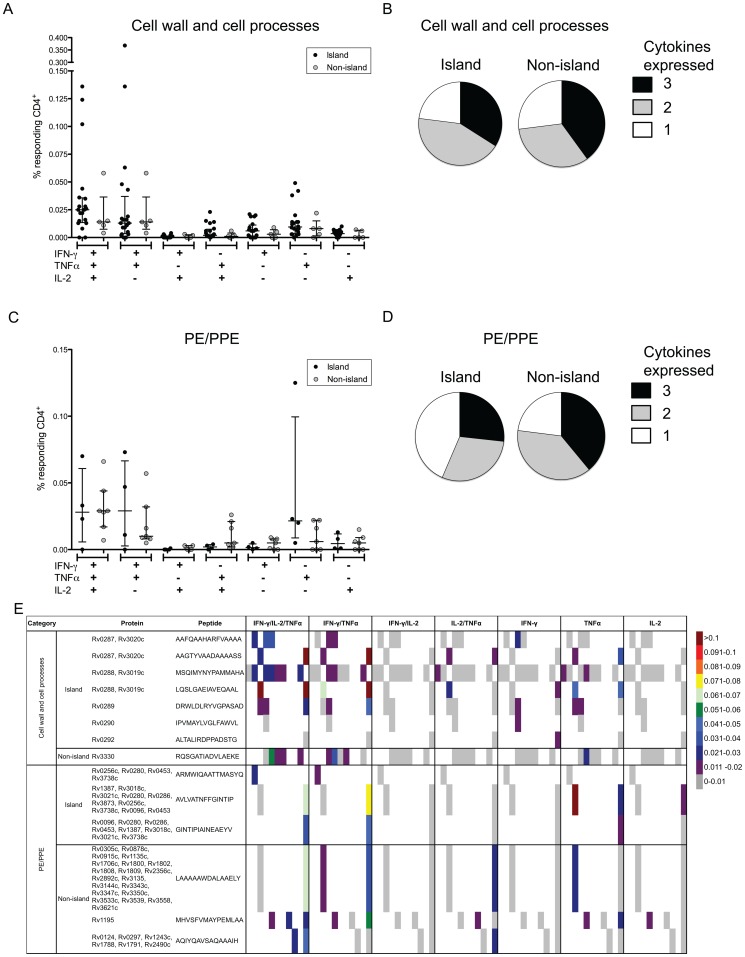
Cell wall/cell processes and PE/PPE specific CD4 T cells have a multifunctional phenotype. Epitope-specific IFN-γ, TNFα and IL-2 production by PBMCs from LTBI donors measured after 6 h peptide stimulation. (A, C) % of responding CD4^+^ expressing each of the seven possible combinations of IFN-γ, TNFα and IL-2 (A) cell wall and cell processes proteins, (C) PE/PPE proteins. Island proteins (black dots) and non-island (grey dots). Each dot represents one donor/epitope combination median ± interquartile range is indicated. (B, D) The fraction of the total cytokine response against (B) cell wall and cell processes, (D) PE/PPE proteins, expressing all 3, 2 or 1 cytokine. (E) Heat-map of each of the seven possible combinations of IFN-γ, TNFα and IL-2 for each individual donor and epitope tested grouped by protein category and island localization. Each column represents one donor. Colors indicate frequency of epitope-specific CD4 T cells, grey is considered negative for indicated cytokine production. Multiple proteins indicate that the peptide sequence is homologous in these proteins.

The frequency of IFN-γ, TNFα, and IL-2 expressing CD4 T cells elicited by proteins from the PE/PPE and cell wall and cell processes category, and from within an island versus non-island, induced similar cytokine expression patterns (Figure 4A and C; gating strategy in Figure S3). The vast majority of CD4^+^ T cells were IFN-γ^+^TNFα^+^IL-2^+^ or IFN-γ^+^TNFα^+^, followed by TNFα^+^ single producing CD4^+^ T cells. To a lesser extent, TNFα^+^IL-2^+^, single IFN-γ^+^, and single IL-2^+^ cells were also detected (Figure 4A and C). Triple cytokine producers were found in 27–40% of cytokine-expressing CD4^+^ T cells, 30–43% expressed any 2 cytokines, and 23–44% produced a single cytokine (Figure 4B and D). We did not observe any donor-, antigen- or epitope-specific pattern of cytokine production (Figure 4E).

### Memory Phenotypes and T Cell Subsets Associated with Different Categories of MTB Antigens

**Figure 5:**
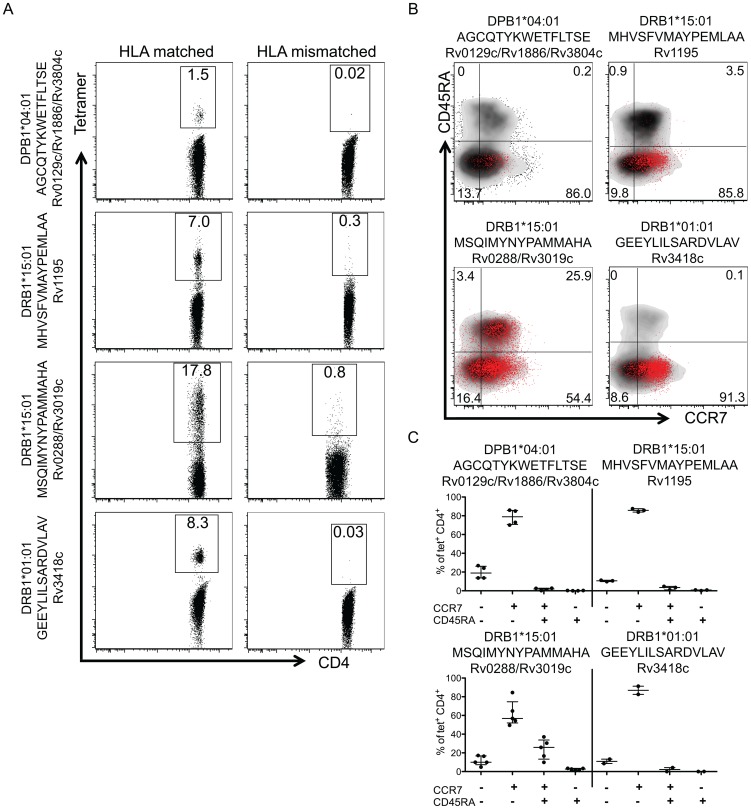
Memory phenotype of MTB-specific CD4 T cells using HLA class II tetramers. (A) HLA class II tetramer stained CD4-purified cells from LTBI donors. Tetramer^+^ cells were isolated following magnetic bead enrichment. Plots are gated on CD4^+^ T cells, and the numbers indicate the percentage of tetramer^+^ cells isolated from each of 4 representative donors CD4^+^ population. DPB1*04:01 AGCQTYKWETFLTSE n = 4 donors, DRB1*15:01 MHVSFVMAYPEMLAA n = 3, DRB1*15:01 MSQIMYNYPAMMAHA n = 5 and DRB1*01:01 GEEYLILSARDVLAV n = 2. (B) Memory phenotype of tetramer^+^ cells for one representative donor per tetramer. Plots are gated on total CD4^+^ T cells (black background) or epitope-specific CD4^+^ T cells (red dots). The numbers represent the percentages of tetramer^+^CD4^+^ T cells in the gate. (C) Scatter plot of the proportion of CCR7^−^CD45RA^−^ (effector memory), CCR7^+^CD45RA^−^ (central memory), CCR7^+^CD45RA^+^ (naïve), and CCR7^−^CD45RA^+^ (effector) CD4^+^ T cells for each tetramer. Each dot represents one donor/tetramer combination, median ± interquartile range is indicated.

CD4^+^ T cells were stained with selected HLA-epitope tetramer reagents and tetramer^+^ cells were enriched [8], [35]. Epitope-specific T cell responses were detected in 16 donors at frequencies 0.25 to 24.3% (median of 3.8, interquartile range 1.5–15.3) for seven different HLA/T cell epitope tetramer combinations (Figure 5A). Only a small number of tetramer-positive cells were detected with the epitope-specific tetramers in donors with a HLA mismatch (Figure 5A), which confirmed that tetramer specificity was derived from the epitope and HLA molecule combination. Epitope tetramer combinations were selected based on the number of donors responding, HLA restriction, and the availability of corresponding reagents for tetramer production. Memory subset phenotypes were determined using Abs to CD45RA and CCR7. Similar to the multifunctionality phenotype, we did not observe any differences in memory phenotype when comparing proteins from within an island vs. non-island (Figure 5B and C). Rv0129c/Rv1886/Rv3804, Rv3418c and Rv1195 epitope-specific tetramer^+^ T cells predominantly consisted of CD45RA^−^CCR7^+^ central memory T cells in all donors analyzed, followed by effector memory (CD45RA^−^CCR7^−^). Percentages ranged between 70.1 and 91.3% (median 85.0, interquartile range (77.7–86.8)) for central memory T cells and 8.6–26.8% (13.3 (10.2–19.0)) for effector memory T cells. Only a minor fraction appeared to be naïve (CCR7^+^CD45RA^+^) or effector T cells (CCR7^−^CD45RA^+^). For Rv0288/Rv3019c the percentages ranged between 49.5 to 84.5% (56.8 (52.0–74.7)) for central memory T cells, 9.8–37.1% (25.9 (13.3–33.8)) for naïve and 4.8–17.2% (10.0 (7.4–16.8)) for effector memory T cells. Again, a minor fraction of the tetramer^+^ cells appeared to be effector T cells (Figure 5B and C).

### The T Cell Response to MTB Is Restricted to a CXCR3^+^CCR6^+^ Memory Subset

**Figure 6:**
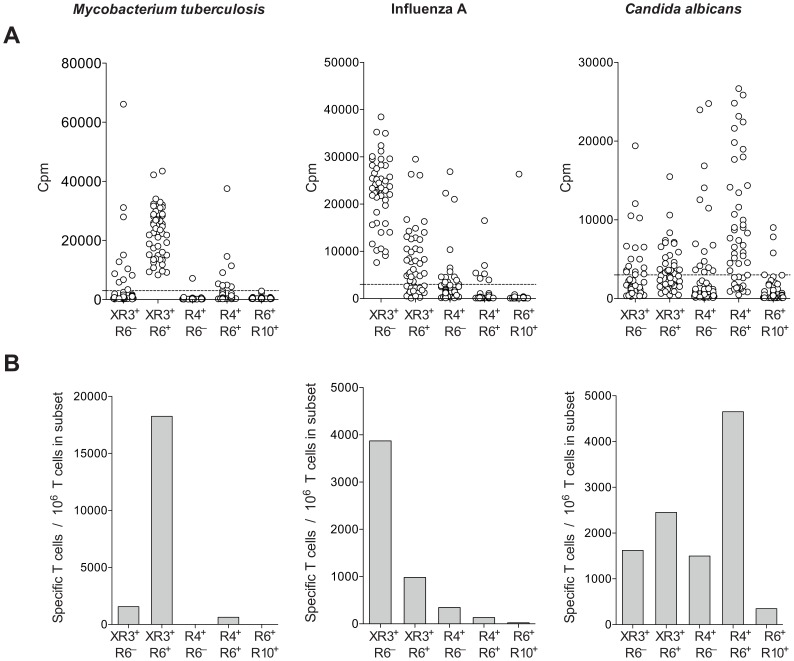
T cell responses to MTB are restricted to a CXCR3^+^CCR6^+^ memory subset. Memory CD4 T cells were sorted into five subsets, 1) CXCR3^+^CCR6^−^, 2) CXCR3^+^CCR6^+^, 3) CCR4^+^CCR6^−^, 4) CCR4^+^CCR6^+^, and 5) CCR6^+^CCR10^+^. The sorted T cells were polyclonally expanded and analyzed for the presence of MTB-, Influenza A- and *C.albicans*-specific T cells by stimulation with whole cell lysates in the presence of autologous monocytes and assessed for ^3^H-thymidine incorporation. Results are from one representative donor of more than 10 tested. (A) Shown is proliferation of individual cultures as cpm. Dotted lines represent the cut-off value. (B) Shown is the estimated frequency of antigen-specific T cells per 10^6^ cells in each Th cell subset for the three organisms used for stimulation.

To measure frequency and distribution of MTB-specific T cells, we used the T cell library method [26]. The majority of epitope-specific tetramer^+^ cells were found to be CD45RA^−^. We therefore stained CD45RA^−^CD25^−^ CD4 T cells from donors latently infected with TB (LTBI) with antibodies against chemokine receptors preferentially expressed on functionally distinct memory T cell subsets [36]. Five Th cell subsets were sorted: 1) CXCR3^+^CCR6^−^; 2) CXCR3^+^CCR6^+^, both enriched in Th1 cells; 3) CCR4^+^CCR6^−^ (Th2); 4) CCR4^+^CCR6^+^ (Th17); and 5) CCR6^+^CCR10^+^ (Th22) [5]. MTB-specific T cells were almost exclusively found in the CXCR3^+^CCR6^+^ subset, while Flu-specific T cells were in the CXCR3^+^CCR6^−^ and CXCR3^+^CCR6^+^ subsets, and *Candida albicans*-specific T cells were most prominent in the CCR4^+^CCR6^+^ subset, enriched in Th17 cells, but positive cultures were also detected in libraries from subsets enriched in Th1, Th2 and Th22 cells (Figure 6A and B). The narrow distribution of antigen-responding T cells in the CXCR3^+^CCR6^+^ subset was peculiar to MTB since *Streptococcus pyogenes-* or *Staphylococcus aureus-*specific T cells were found in both CXCR3^+^CCR6^+^ and CCR4^+^CCR6^+^ subsets (not shown).

**Figure 7:**
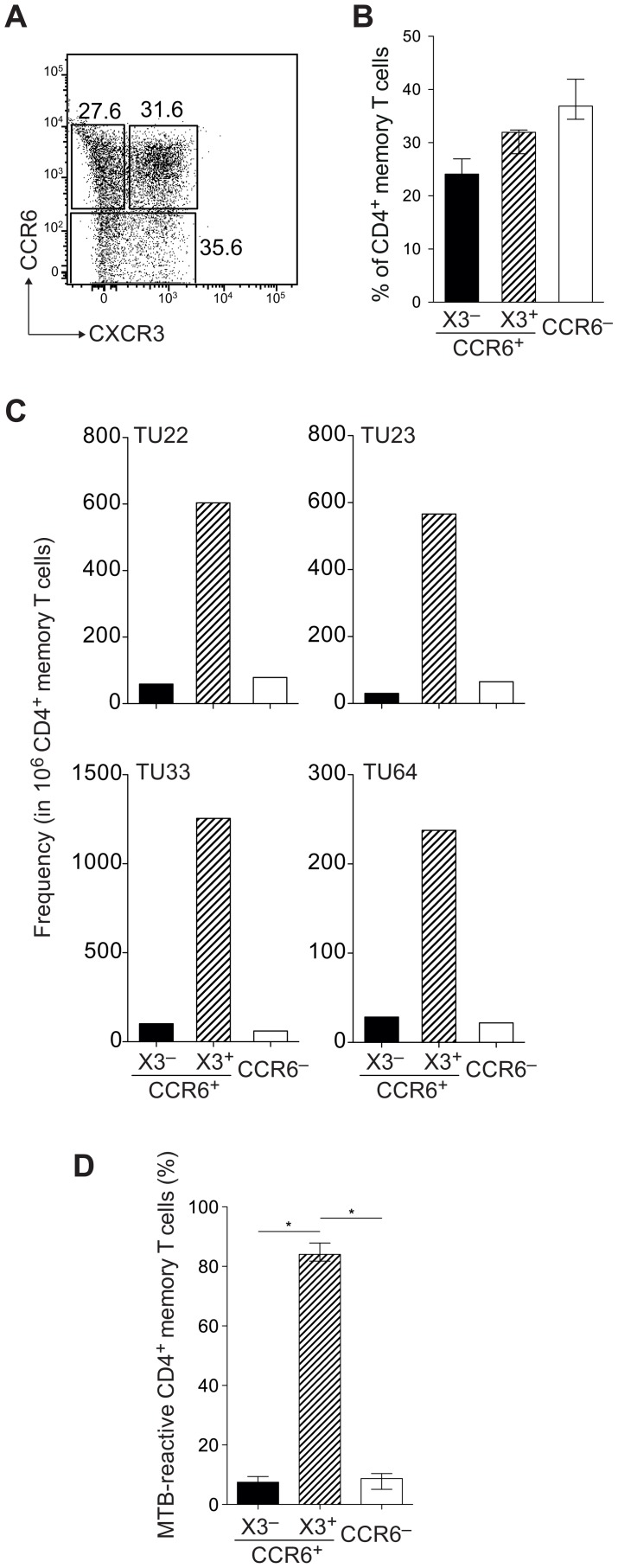
The T cell response to MTB is restricted to a CXCR3^+^CCR6^+^ memory subset. (A, B) Three CD45RA^−^CD25^−^CD4^+^ memory T cell subsets from four LTBI donors were sorted; 1) CCR6^+^CXCR3^−^; 2) CCR6^+^CXCR3^+^; and 3) CCR6^−^. (A) Representative dot plot from one donor; (B) Median percentages of the T cell subsets on total CD4^+^ memory T cells. Error bars indicate interquartile range (n = 4). (C) T cell libraries were set up from the sorted subsets by polyclonal stimulation and expansion for 3–4 weeks. Libraries were analyzed by stimulation with autologous monocytes with or without MTB whole cell lysate and proliferative response was measured by ^3^H-thymidine incorporation. Shown is the estimated frequency of MTB-specific T cells per 10^6^ CD4 memory T cells for LTBI donors. (D) Distribution of MTB-specific T cells in the three memory T cell subsets. Data represent median ± interquartile range from four donors. Mann Whitney test, *, *p*<0.05.

Based on these results, we sorted three memory CD4 Th cell subsets (Figure 7A and B): 1) CCR6^+^CXCR3^−^, accounting for 24.1% (21.8–27.0) (median (interquartile range), n = 4) of the memory CD4^+^ T cell pool; 2) CCR6^+^CXCR3^+^ (32.0% (28.0–32.4)) and 3) CCR6^−^ (37.0% (34.4–42.0)). For each donor a T cell library of 288 cultures was established. MTB-responding T cells were highly enriched in cultures derived from the CCR6^+^CXCR3^+^ T cell subset, and present at much lower frequency in the CCR6^+^CXCR3^−^ and the CCR6^−^ subsets (Figure 7C). This pattern of distribution was remarkable consistent: in all 4 donors analyzed more than 80% of the MTB-reactive memory CD4 T cell response resided in the CXCR3^+^CCR6^+^ subset (Figure 7D).

**Figure 8:**
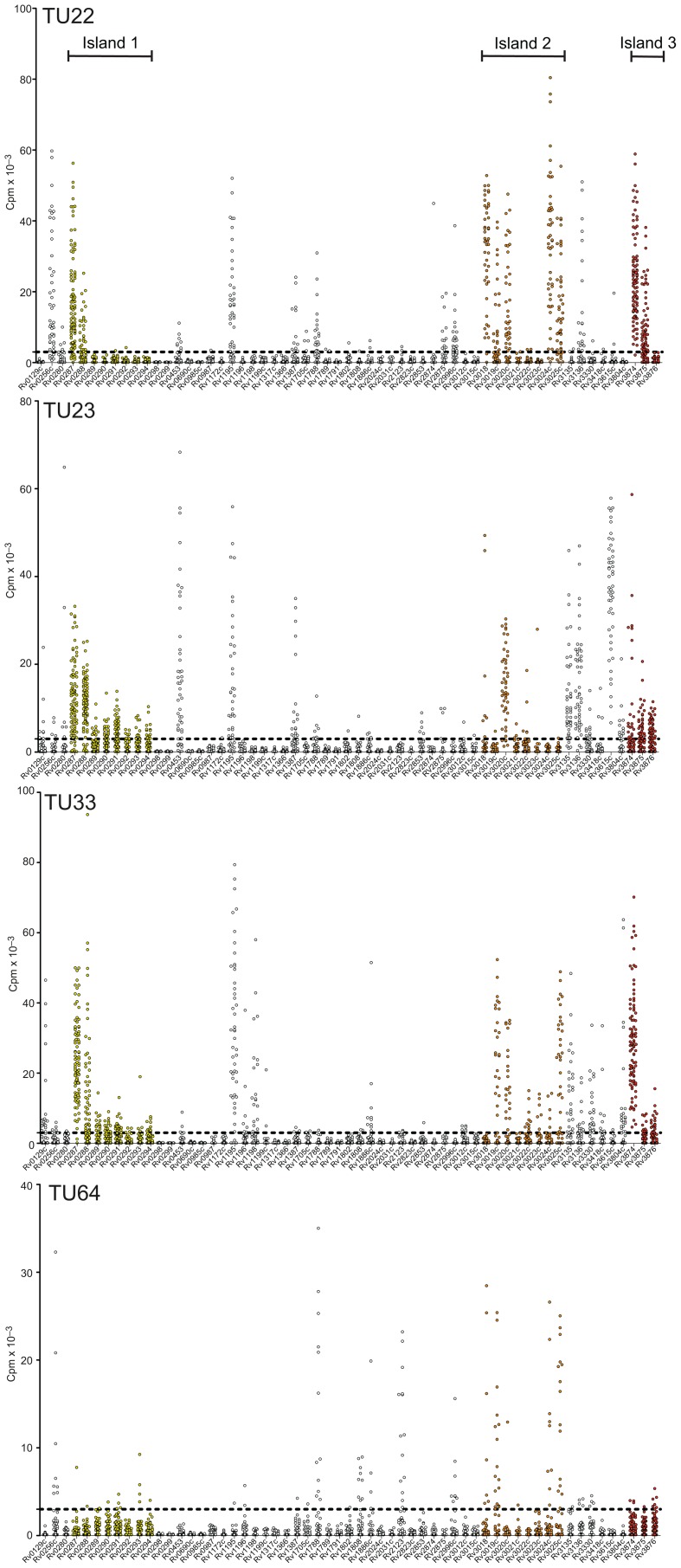
The T cell library approach complements the ex vivo IFN-γ ELISPOT assay. CCR6^+^CXCR3^+^ T cell libraries were set up for 4 representative donors. The sorted T cells were polyclonally expanded and analyzed for the presence of antigen-specific T cells by stimulation with peptide pools and measurement of ^3^H-thymidine incorporation. Shown is proliferation (cpm) of individual cultures from 4 different donors. Dotted lines represent the cut-off value. Response to antigens within genomic islands is shown in yellow, orange, and red; response to antigens outside antigenic islands is shown in white. Antigenic islands are indicated by capped lines.

Next, we set up T cell libraries from 4 representative donors and the CXCR3^+^CCR6^+^ subset were directly stimulated, after expansion, with 59 representative peptide pools. The results of this analysis are shown in figure 8. Using this approach we were able to demonstrate that the results obtained with the MTB lysate also extended to responses specific for the various epitopes, and to confirm with a complementary approach the results of the ex vivo IFN-γ ELISPOT analysis utilizing the library of predicted HLA class II binding epitopes.

## Discussion

Individual MTB proteins have been studied to identify novel vaccine candidates, with several studies focused on culture filtrate proteins [6], [7]. Other studies utilized bioinformatic approaches to select a subset of genes as antigen candidates [37], [38]. However, the lack of a true genome-wide characterization has hindered a complete understanding of the mechanisms and specificity of the immune response to MTB.

This study provides the first in-depth truly genome-wide description of human T cell responses to MTB. We characterized and isolated T cells directly ex vivo, thus avoiding biases introduced as a result of *in vitro* restimulation and expansion of T cells before analysis. This approach should be generally applicable to the study of immunity to other complex pathogens. The HLA alleles were chosen to allow coverage of the most frequent DP, DQ and DR specificities in the general population [39]. However, we readily acknowledge that this selection has potential limitations and may bias the results toward the epitopes recognized by these alleles.

In terms of T cells recognizing MTB we found that the T cell response to MTB antigens in LTBI donors is strongly biased towards a subset of CXCR3^+^ Th1 cells that co-express CCR6 [4]. Interestingly, this narrow distribution was only seen for MTB and not other pathogens such as *S. pyogenes* and *C. albicans* within the same donor. The origin of CCR6^+^ Th1 cells and their differentiation requirements remains to be defined; they may represent a separate Th1 lineage, or they may differentiate from plastic CCR6^−^ Th1 cells or CCR6^+^ Th17 cells [40]. Future studies will examine whether this highly focused response is key to MTB containment by examining patients who remain healthy vs. patients who progress to active disease.

Striking levels of heterogeneity of responses were detected. This expands previous observations using smaller subsets of antigens [6], [41], and a genome-wide screen of antibody responses [28]. The observed heterogeneity might reflect differences in MTB strains, bacillary load, and metabolic state, resulting in qualitative or quantitative differences in antigen expression [42], [43]. In any case, since natural immunity to MTB is multiepitopic and multiantigenic, and more than 80 antigens are necessary to capture 80% of the T cell response, vaccination strategies including one or a few antigens are unlikely to replicate natural immunity. Likewise, monitoring the immune response to one or a few antigens in the setting of clinical trials might yield a severely incomplete and biased picture of immune reactivity.

Several antigens from the DosR regulon, as well as resuscitation- and reactivation-associated antigens have been described as preferentially recognized by individuals with latent infection using long-term T cell cultures [44], [45]. We observed reactivity to two of these proteins, Rv2031c (2 donors) and Rv2627c (1 donor), and no significant association with proteins from the DosR regulon or latency-associated antigens, similar to previous observations [46].

Numerous tuberculosis vaccine candidates are currently in clinical trials, these candidates are based on 11 MTB antigens, 7 of which were prevalently recognized in this study; Rv3804c in MVA85A [47] and Aeras-402 [48], Rv1886c in Aeras-402, H1 [49] and HyVac4 [50], Rv0288 in Aeras-402 and HyVac4, Rv3875 in H1 and H56 [51], Rv1196 in Mtb72f/AS02A [52], Rv2608 and Rv3619 in ID93 [53]. Of the remaining 4 antigens Rv3620c in ID93 was also recognized whereas Rv2660c in H56, Rv0125 in Mtb72f/AS02A and Rv1813c in ID93 were not.

We identified three antigenic islands within the MTB genome map as main determinants of immunodominance. Remarkably, the majority of the novel antigens identified are associated (contained within or in close proximity to) these islands, which all contain Esx protein pairs and PE/PPE proteins, and are part of a putative secretory system. Our analysis demonstrated that these factors synergistically contribute to determining immunodominance and confirms the importance of PE/PPE and Esx proteins, but suggests that their immunodominance is perhaps mostly determined by their location within these antigenic islands.

Two main hypotheses can be put forth to explain the mechanism by which these features determine immunodominance. First, secreted proteins may act as decoys to divert the immune response from recognizing nonsecreted MTB proteins [13], thus favoring bacterial persistence. The second hypothesis envisions that antigenic islands are dominant because they are intrinsically immunogenic, and because they perform key biological functions necessary to maintain MTB persistence.

The decoy hypothesis has two predicated features; either secreted proteins result in diversion of the immune system from the bacteria itself, or the decoy effect is achieved by inducing an immune response to decoy antigens that are functionally distinct from non-decoy antigens. In the first case, we note that both secreted proteins and proteins involved in the secretion apparatus are equally recognized. Indeed, immune reactivity towards proteins involved in the secretion system apparatus has previously been described for T3SS and inflammasome activation by flagellin and the T3SS rod proteins [54], [55]. Furthermore, we were unable to detect a functionally distinct immune response in terms of multifunctionality, memory phenotype and T cell subsets, and independent of island vs. non-island localization and secretion status of the antigen recognized. Taken together, these observations argue against the decoy hypothesis. T cells that secrete multiple cytokines are a potential correlate of protection, but have also been implicated in pathology [31], [32], [33], [34]. Whatever their role might be, the majority of epitope-specific CD4^+^ T cell responses were multifunctional, with no differences between antigens from islands vs. non-islands, and between the PE/PPE vs. cell wall and cell processes categories. In terms of T cell phenotypes and T cell subsets a similar picture emerged, with epitope specific CD4^+^ T cells being predominantly CD45RA^−^CCR7^+^ central memory cells, in agreement with previous studies [26]. For some epitope specific CD4^+^ T cells a large fraction were CD45RA^+^CCR7^+^, a phenotype traditionally regarded as naïve. Such T cells have previously been reported [56], [57], and might reflect early differentiation into antigen-specific cells. Additional studies would be required to investigate this further.

The available data favors the second hypothesis, that the three antigenic islands are dominant because they perform key biological functions and are necessary to maintain MTB persistence. The most prevalently recognized island is Esx-3, which is controlled by the iron-dependent regulator IdeR and the zinc uptake regulator Zur [58], [59], suggesting its involvement in fundamental biological processes such as metal iron homeostasis. In addition, Esx-3 is essential for *in vitro* growth, and is conserved in a wide range of mycobacterial species [20], [60]. Furthermore, the Esx-3 system contributes to immune protection against MTB challenge in mice of the IKEPLUS strain [61] in a HLA class II dependent fashion.

Genes from island 2 are, like island 1 (Esx-3), regulated by Zur [58], providing a possible functional link between them. While island 2 is not part of an Esx secretion system *per se*, it is believed to originate from a duplication of the Esx-3 system [19]. Esx-1 and Esx-3 also appear to be linked, since Rv3873 interacts with Rv0288 [62].

Secretion systems similar to the T7SS associated with two of the three antigenic islands are also found in other bacteria, such as *Listeria monocytogenes*, *S. aureus*, and *Bacillus anthrax* [63]. Secretion of the substrates from T7SS are not dependent on interaction with host cells, unlike other bacterial secretion systems such as T3SS and T4SS, which are switched on upon host-cell contact. This suggests that T7SS, while essential for pathogenicity, may fulfill more general physiological roles than strictly host-cell oriented functions.

This study was completed in a non-TB-endemic population. Ongoing studies include a larger study population from different ethnicities and geographic locations, as well as patients with different disease states and BCG vaccination status. This will provide answers for different HLA phenotypes, as well as whether patients from an endemic area or with different disease states show a different recognition pattern.

In conclusion, this study describes the immunological footprint of MTB CD4 T cell recognition to an unprecedented level of detail. The high throughput cellular screens utilized here to analyze the human immune response to MTB provides information on the specificity, frequency and class of memory T cells, as well as on the individual variability in magnitude and quality of the response. As a result, 34 novel antigens and three broadly immunodominant antigenic islands were defined. The study of the class of proteins recognized, together with the phenotype of responding T cells, disproves the notion that responses against secreted antigens are a decoy utilized to favor bacterial persistence, and rather suggest that these proteins, together with those that are part of their general secretion apparatus, are targeted by fully functional T cell responses. More broadly, this study provides proof of principle of how such high throughput techniques can be applied to other complex pathogen systems. In terms of potential practical applications, the novel T cell antigens identified could be of potential use for diagnostic or vaccine purposes. Indeed, the heterogeneity of responses demonstrated herein suggests that a too narrow focus for vaccine evaluations will not replicate natural immunity. Finally, the antigens and epitopes identified can also be used as tools for identifying biomarkers to provide correlates of risk for, or protection against, tuberculosis disease.

## Materials and Methods

### Ethics Statement

Research conducted for this study was performed in accordance with approvals from the Institutional Review Board at the La Jolla Institute for Allergy and Immunology. All participants provided written informed consent prior to participation in the study.

### Study Subjects

Leukapheresis samples from 28 adults with LTBI and 28 control donors were obtained from the University of California, San Diego Antiviral Research Center clinic (age range 20–65 years). Subjects had a history of a positive tuberculin skin test (TST). LTBI was confirmed by a positive QuantiFERON-TB Gold In-Tube (Cellestis), as well as a physical exam and/or chest X-ray that was not consistent with active tuberculosis. None of the study subjects endorsed vaccination with BCG, or had laboratory evidence of HIV or Hepatitis B. The control donors had a negative TST, as well as a negative QuantiFERON-TB. Approval for all procedures was obtained from the Institutional Review Board (FWA#00000032) and informed consent was obtained from all donors.

### Bioinformatic Analyses

Proteins from the 21 MTB genome projects available from the NCBI Protein database were downloaded into an in-house MySQL database. Of these, 5 were complete (CDC1551, F11, H37Ra, H37Rv, KZN 1435) and 16 were draft assemblies (Table S1). The protein sequences were parsed into all possible 15mer peptides (n = 1,568,148), for each of which binding to 22 different HLA DR, DP and DQ class II alleles most commonly expressed in the general population (Table S2) was predicted using the IEDB HLA class II ‘consensus’ prediction method [64]. The sequences of the H37Rv strain were used as a reference sequence. For each H37Rv protein, alignments were made of all orthologs identified in other genomes, as determined by a BLAST search. Because of the overall high sequence conservation among the proteins from all the 21 genomes, 1,220,829 (91.4%) of 15mers were completely conserved among all of the strains. For each protein, the best-predicted binders, as ranked by consensus percentile, were selected for synthesis. In order to ensure coverage of each of the proteins, the number of peptides selected per protein was no less than 2 and no more than 10, depending upon protein length (18,950 peptides). Any variants among the orthologs at the selected positions were also selected (1,660), for a total of 20,610 peptides.

### Peptides

Sets of 15-mer peptides synthesized by Mimotopes (Victoria, Australia) and/or A and A (San Diego) as crude material on a small (1 mg) scale were combined into pools of 20 peptides. Peptides utilized for tetramers were synthesized as purified material (>95% by reversed phase HPLC). The IEDB submission number for the peptides is 1000505.

### PBMC Isolation

PBMCs were obtained by density gradient centrifugation (Ficoll-Hypaque, Amersham Biosciences) from 100 ml of leukapheresis sample, according to manufacturer's instructions. Cell were suspended in fetal bovine serum (Gemini Bio-products) containing 10% dimethyl sulfoxide, and cryo-preserved in liquid nitrogen.

### T Cell Library

CD4 T cells were isolated from PBMCs by positive selection with microbeads (Miltenyi Biotec). Memory CD4^+^ T cell subsets were sorted with a FACSAria (BD Biosciences) to over 98% purity excluding CD45RA^+^, CD25^+^, CD8^+^, CD19^+^, and CD56^+^ cells. Antibodies used for positive selection were: anti-CCR6-PE or biotinylated (11A9; BD Biosciences) followed by streptavidin-allophycocyanin (APC) (Invitrogen) or streptavidin-APC-cyanine7 (APC-Cy7) (BD Biosciences); anti-CCR10-PE (314305, R&D Systems), anti-CCR4-PE-Cy7 (1G1; BD Pharmingen) and anti-CXCR3-APC (1C6; BD Pharmigen). Cells were cultured in RPMI 1640 medium supplemented with 2 mM glutamine, 1% (vol/vol) nonessential amino acids, 1% (vol/vol) sodium pyruvate, penicillin (50 U/ml), streptomycin (50 µg/ml) (all from Invitrogen) and 5% heat-inactivated human serum (Swiss Red Cross). T cells (1,000 cells/well) were stimulated polyclonally with 1 µg/ml PHA (Remel) in the presence of irradiated (45 Gy) allogeneic feeder cells (1.0×10^5^ per well) and IL-2 (500 IU/ml) in a 96-well plate format and T cell lines were expanded as previously described [26]. Library screening was performed at day 14–21 by culturing extensively washed T cells (∼2.5×10^5^/well) with autologous monocytes (2.5×10^4^), either unpulsed or pulsed for 3 h with MTB whole cell lysate (5 µg/ml, BEI Resources) or control antigens. In some experiments, T cells were cultured with peptide pools (2 µg/ml). Proliferation was measured on day 2–3 after 16 h incubation with 1 µCi/ml [methyl-^3^H]-thymidine (Perkin Elmer). Precursor frequencies were calculated based on numbers of negative wells according to the Poisson distribution and expressed per million cells.

### Ex Vivo IFN-γ ELISPOT Assay

PBMCs incubated at a density of 2×10^5^ cells/well were stimulated with peptide pools (5 µg/ml) or individual peptides (10 µg/ml), PHA (10 µg/ml) or medium containing 0.25% DMSO (corresponding to percent DMSO in the pools/peptides, as a control) in 96-well plates (Immobilon-P; Millipore) coated with 10 µg/ml anti-IFN-γ (AN18; Mabtech). Each peptide or pool was tested in triplicate. After 20 h incubation at 37°C, wells were washed with PBS/0.05% Tween 20 and incubated with 2 µg/ml biotinylated anti-IFN-γ (R4-6A2; Mabtech) for 2 h. The spots were developed using Vectastain ABC peroxidase (Vector Laboratories) and 3-amino-9-ethylcarbazole (Sigma-Aldrich) and counted by computer-assisted image analysis (KS-ELISPOT reader, Zeiss). Responses were considered positive if the net spot-forming cells (SFC) per 10^6^ were ≥20, the stimulation index ≥2, and p<0.05 (Student's t-test, mean of triplicate values of the response against relevant pools or peptides vs. the DMSO control). For experiments utilizing depletion of CD4^+^ or CD8^+^ T cells, these cells were isolated by positive selection (Miltenyi Biotec) and effluent cells (depleted cells) were used for experiments.

The response frequency was calculated by dividing the no. of donors responding with the no. of donors tested. The magnitude of response (total SFC) was calculated by summation of SFC from responding donors.

### Intracellular Cytokine Staining

PBMCs were cultured in the presence of 5 µg/ml MTB peptide and 4 µl/ml Golgiplug (BD Biosciences) in complete RPMI medium at 37°C in 5% CO_2_. Unstimulated PBMCs were used to assess nonspecific/background cytokine production. After 6 h, cells were harvested and stained for cell surface antigens CD4 (anti-CD4-PerCPCy5.5, OKT-4) and CD3 (anti-CD3-EFluor450, UCHT1). After washing, cells were fixed and permeabilized, using a Cytofix/Cytoperm kit (BD Biosciences) and then stained for IFN-γ (anti-IFN-γ-APC, 4S.B3), TNFα (anti-TNFα-FITC, MAb11) and IL-2 (anti-IL-2-PE, MQ1-17H12). All antibodies were from eBioscience. Samples were acquired on a BD LSR II flow cytometer. The frequency of CD4^+^ T cells responding to each MTB peptide was quantified by determining the total number of gated CD4^+^ and cytokine^+^ cells and background values subtracted (as determined from the medium alone control) using FlowJo software (Tree Star). A cut-off of 2 times the background was used. Combinations of cytokine producing cells were determined using Boolean gating in FlowJo software.

### Tetramer Staining

HLA class II tetramers conjugated using PE labeled streptavidin were provided by the Tetramer Core Laboratory at Benaroya Research Institute. CD4 T cells were purified using the Miltenyi T cell isolation kit II according to manufacturer's instructions. Purified cells (∼10×10^6^) were incubated in 0.5 ml PBS containing 0.5% BSA and 2 mM EDTA pH 8.0 (MACS buffer) with a 1∶50 dilution of class II tetramer for 2 h at room temperature. Cells were then stained for cell surface antigens using anti-CD4-FITC (OKT-4), anti-CD3-Alexa Fluor 700 (OKT3), anti-CCR7-PerCPEFluor710 (3D12), anti-CD45RA-EFluor450 (HI100) (all from EBioscience) and Live/Dead Yellow (Life Technologies) to exclude dead cells. Tetramer-specific T cell populations were enriched by incubating cells with 50 µl of anti-PE microbeads (Miltenyi Biotech) for 20 min at 4°C. After washing, cells were resuspended in 5 ml MACS buffer and passed through a magnetized LS column (Miltenyi Biotec). The column was washed three times with 3 ml of MACS buffer, and after removal from the magnetic field, cells were collected with 5 ml of MACS buffer. Samples were acquired on an BD LSR II flow cytometer and analyzed using FlowJo software.

### Antigen and IEDB Analysis

The identified epitopes were compared for sequence homology and the weakest epitopes sharing >90% homology were eliminated. The epitopes were mapped to the H37Rv genome allowing 1 substitution per peptide, to identify antigens. IEDB queries utilized criteria matching the experimental study (organism; MTB, host organism; human, latent disease, ex vivo, HLA class II). Epitopes were then mapped as above. To capture the most frequently recognized antigens the response frequency score (no. donors responded – Square root of no. donors responded/no. donors tested), was utilized [65].

## Supporting Information

Figure S1 **Experimental design.** Summary of the steps involved in the antigen identification pipeline, showing number of genomes, 15-mer peptides and selected peptides.
(EPS)

Figure S2 **Novelty of the antigens identified as a source of CD4 epitopes in humans.** (A) Comparison with IEDB and literature, antigens were divided into four categories; novel, targets of CD4 T cells, CD8 T cells or undefined T cell type. 41% of defined antigens are novel. (B) Overlap of antigens described in this study with antigens described as sources of HLA class I restricted epitopes in the IEDB. (C) Overlap of antigens described in this study with antigens described as serologically reactive by Kunnath-Velayudhan et al. p-values calculated using a Chi-square test.
(EPS)

Figure S3 **Gating strategy for multifunctionality analysis.** Cells were first gated based on forward vs. side-scatter, then CD3 vs. CD4 and finally for each cytokine (IFN-γ, TNFα, IL-2). Gates for each cytokine were based on the negative control and they were used for subsequent Boolean gating.
(EPS)

Table S1 **Summary of MTB genomes used for peptide predictions.**
(DOC)

Table S2 **Haplotype and phenotype frequencies of HLA class II alleles used for predictions.**
(DOC)

Table S3 **Summary of epitope characteristics.**
(XLS)

Table S4 **Summary of characteristics of antigens recognized by more than 10% of LTBI donors according to magnitude of response.**
(XLS)
