# Supplementary material for: Memory T Cells in Latent Mycobacterium tuberculosis Infection Are Directed against Three Antigenic Islands and Largely Contained in a CXCR3+CCR6+ Th1 Subset
Source: PLoS Pathog. 2013 Jan 24;9(1):e1003130. doi: 10.1371/journal.ppat.1003130 (PMC3554618; doi:10.1371/journal.ppat.1003130)
Supplement: Table S1 — Summary of MTB genomes used for peptide predictions. (DOC) [file ppat.1003130.s004.doc]

**Table S1. Summary of MTB genomes used for peptide predictions**

| **GenBank accession no.** | **Organism** | **No. protein sequences^a^** | **No. unique 15-mer peptides in genome** |
| --- | --- | --- | --- |
| **NC_000962** | Mycobacterium tuberculosis H37Rv | 3,988 | 1,258,608 |
| **NC_002755** | Mycobacterium tuberculosis CDC1551 | 4,189 | 1,252,098 |
| **NC_009525** | Mycobacterium tuberculosis H37Ra | 4,034 | 1,262,786 |
| **NC_009565** | Mycobacterium tuberculosis F11 | 3,941 | 1,261,978 |
| **NC_012943** | Mycobacterium tuberculosis KZN 1435 | 4,059 | 1,265,498 |
| **NZ_ABGN00000000** | Mycobacterium tuberculosis KZN 605 | 3,972 | 1,097,739 |
| **NZ_AAKR00000000** | Mycobacterium tuberculosis C | 3,508 | 1,060,472 |
| **NZ_AASN00000000** | Mycobacterium tuberculosis str. Haarlem | 3,596 | 1,108,161 |
| **NZ_AAYK00000000** | Mycobacterium tuberculosis H37Ra | 4,438 | 1,133,553 |
| **NZ_ABGL00000000** | Mycobacterium tuberculosis KZN 4207 | 4,068 | 1,147,062 |
| **NZ_ABLL00000000** | Mycobacterium tuberculosis 94_M4241A | 4,232 | 1,166,312 |
| **NZ_ABLM00000000** | Mycobacterium tuberculosis 02_1987 | 4,266 | 1,181,241 |
| **NZ_ABLN00000000** | Mycobacterium tuberculosis T92 | 4,254 | 1,085,346 |
| **NZ_ABOV00000000** | Mycobacterium tuberculosis EAS054 | 4,101 | 1,167,286 |
| **NZ_ABOW00000000** | Mycobacterium tuberculosis T85 | 4,206 | 1,130,366 |
| **NZ_ABQG00000000** | Mycobacterium tuberculosis GM 1503 | 4,116 | 1,091,459 |
| **NZ_ABQH00000000** | Mycobacterium tuberculosis T17 | 4,254 | 1,116,545 |
| **NZ_ABVM00000000** | Mycobacterium tuberculosis '98-R604 INH-RIF-EM' | 4,112 | 1,174,559 |
| **NZ_ACHO00000000** | Mycobacterium tuberculosis T46 | 4,134 | 1,155,871 |
| **NZ_ACHP00000000** | Mycobacterium tuberculosis CPHL_A | 4,140 | 1,196,800 |
| **NZ_ACHQ00000000** | Mycobacterium tuberculosis K85 | 4,196 | 1,201,360 |

^a^) Data available in GenBank as of December 2009
