# Supplementary material for: Memory T Cells in Latent Mycobacterium tuberculosis Infection Are Directed against Three Antigenic Islands and Largely Contained in a CXCR3+CCR6+ Th1 Subset
Source: PLoS Pathog. 2013 Jan 24;9(1):e1003130. doi: 10.1371/journal.ppat.1003130 (PMC3554618; doi:10.1371/journal.ppat.1003130)
Supplement: Table S2 — Haplotype and phenotype frequencies of HLA class II alleles used for predictions. (DOC) [file ppat.1003130.s005.doc]

Table S2. Haplotype and phenotype frequencies of HLA class II alleles used for predictions

| **Locus** | **Allele** | **Percent of haplotypes** | **Phenotype frequency** |
| --- | --- | --- | --- |
| DRB1 | DRB1*01:01 | 2.8 | 5.4 |
|  | DRB1*03:01 | 7.1 | 13.7 |
|  | DRB1*04:01 | 2.3 | 4.6 |
|  | DRB1*04:05 | 3.1 | 6.2 |
|  | DRB1*07:01 | 7.0 | 13.5 |
|  | DRB1*08:02 | 2.5 | 4.9 |
|  | DRB1*11:01 | 6.1 | 11.8 |
|  | DRB1*12:01 | 2.0 | 3.9 |
|  | DRB1*13:02 | 3.9 | 7.7 |
|  | Total | 36.8 |  |
| DRB3/4/5 | DRB3*01:01 | 14.0 | 26.1 |
|  | DRB4*01:01 | 23.7 | 41.8 |
|  | DRB5*01:01 | 8.3 | 16.0 |
|  | Total | 46.0 |  |
| DQA1/DQB1 | DQA1*05:01/DQB1*02:01 | 5.8 | 11.3 |
|  | DQA1*05:01/DQB1*03:01 | 19.5 | 35.1 |
|  | DQA1*03:01/DQB1*03:02 | 10.0 | 19.0 |
|  | DQA1*04:01/DQB1*04:02 | 6.6 | 12.8 |
|  | DQA1*01:01/DQB1*05:01 | 7.6 | 14.6 |
|  | Total | 49.5 |  |
| DPB1 | DPA1*02:01/DPB1*01:01 | 8.4 | 16.0 |
|  | DPA1*01:03/DPB1*02:01 | 9.2 | 17.5 |
|  | DPA1*01:03/DPB1*04:01 | 20.1 | 36.2 |
|  | DPA1*03:01/DPB1*04:02 | 23.6 | 41.6 |
|  | DPA1*02:01/DPB1*05:01 | 11.5 | 21.7 |
|  | Total | 72.8 |  |

Average haplotype and phenotype frequencies for individual alleles are based on data available at dbMHC. dbMHC data considers prevalence in Europe, North Africa, North-East Asia, the South Pacific (Australia and Oceania), Hispanic North and South America, American Indian, South-East Asia, South-West Asia, and Sub-Saharan Africa populations. DP, DRB1 and DRB3/4/5 frequencies consider only the beta chain frequency, given that the DR alpha chain is largely monomorphic, and that differences in DPA are not considered to significantly influence binding. Frequency data are not available for DRB3/4/5 alleles, however, because of linkage with DRB1 alleles, coverage for these specificities may be assumed as follows: DRB3 with DR3, DR11, DR12, DR13 and DR14; DRB4 with DR4, DR7 and DR9; DRB5 with DR15 and DR16. Specific allele frequencies at each B3/B4/B5 locus is based on published associations with various DRB1 alleles, and assumes only limited variation at the indicated locus.
